# Differential Role of PTEN Phosphatase in Chemotactic Growth Cone Guidance*[Fn FN2]

**DOI:** 10.1074/jbc.C113.487066

**Published:** 2013-06-17

**Authors:** Steven J. Henle, Lucas P. Carlstrom, Thomas R. Cheever, John R. Henley

**Affiliations:** From the ‡ Department of Neurologic Surgery,; § Medical Scientist Training Program, and; ¶ Department of Physiology and Biomedical Engineering, Mayo Clinic, Rochester, Minnesota 55905

**Keywords:** Axon, BDNF, Chemotaxis, Integrin, Neurite Outgrowth, Neurodevelopment, Phosphatidylinositol Signaling, PTEN, Growth Cone Guidance, Myelin-associated Glycoprotein

## Abstract

Negatively targeting the tumor suppressor and phosphoinositide phosphatase PTEN (phosphatase and tensin homologue) promotes axon regrowth after injury. How PTEN functions in axon guidance has remained unknown. Here we report the differential role of PTEN in chemotactic guidance of axonal growth cones. Down-regulating PTEN expression in *Xenopus laevis* spinal neurons selectively abolished growth cone chemorepulsion but permitted chemoattraction. These findings persisted during cAMP-dependent switching of turning behaviors. Live cell imaging using a GFP biosensor revealed rapid PTEN-dependent depression of phosphatidylinositol 3,4,5-trisphosphate levels in the growth cone induced by the repellent myelin-associated glycoprotein. Moreover, down-regulating PTEN expression blocked negative remodeling of β1-integrin adhesions triggered by myelin-associated glycoprotein, yet permitted integrin clustering by a positive chemotropic treatment. Thus, PTEN negatively regulates growth cone phosphatidylinositol 3,4,5-trisphosphate levels and mediates chemorepulsion, whereas chemoattraction is PTEN-independent. Regenerative therapies targeting PTEN may therefore suppress growth cone repulsion to soluble cues while permitting attractive guidance, an essential feature for re-forming functional neural circuits.

## Teaser

**Background:** The phosphatase PTEN is implicated in suppressing neuroregeneration following injury.

**Results:** Chemorepulsion of axons by distinct cues, but not chemoattraction, correlates with PTEN activity, depression of phosphatidylinositol signaling, and remodeling of integrin adhesions.

**Conclusion:** PTEN mediates chemorepulsion selectively.

**Significance:** Suppressing PTEN activity may block repulsion by negative cues after injury while permitting attractive guidance of regenerating axons.

## Introduction

Growing axons make selective pathway choices to develop specific synaptic connections during development and regeneration (1, 2). Extrinsic cues in the microenvironment guide this pathfinding by exerting either attractive or repulsive actions on the axonal growth cone, mediated locally by cytoplasmic second messengers (3–8). These second messengers in turn regulate output processes such as actin dynamics (9, 10) and remodeling of integrin adhesions (11, 12) that steer the growth cone.

We reported previously that, similar to positive chemotaxis of amoeboid cells, local elevation of the second messenger phosphatidylinositol 3,4,5-trisphophate (PIP_3_)^3^ mediates growth cone chemoattraction (13). However, how changes in PIP_3_ levels may direct growth cone chemorepulsion is unknown. Evidence from *Dictyostelium* indicates that application of an artificial chemorepulsive cue locally depresses PIP_3_ levels (14). This local decrease in PIP_3_ likely involves the actions of the tumor suppressor phosphatase and tensin homologue (PTEN), which dephosphorylates PIP_3_ and facilitates the establishment of polarity during amoeboid chemotaxis (15, 16).

Intriguingly, modulation of PTEN function has shown promise for promoting neural regeneration after injury, where the release of axon outgrowth inhibitory cues such as myelin-associated glycoprotein (MAG) is thought to be a significant impediment to functional regeneration (17–20). Depletion of PTEN in the mammalian central nervous system (CNS) for example results in a dramatic increase in regenerative axon outgrowth *in vivo* (21–23). In addition, down-regulating PTEN in cultured neurons partially blocks inhibition of neurite outgrowth by MAG (24). Based on these foundational studies, we tested the hypothesis that PTEN functions in bidirectional growth cone chemotaxis by locally mediating the actions of guidance cues on PIP_3_ second messenger signals and downstream effectors.

Here we demonstrate the differential role of PTEN in growth cone chemorepulsion, but not chemoattraction. The selective function of PTEN in chemorepulsion was irrespective of the guidance cue, as revealed by directly manipulating cAMP signaling activity to switch growth cone turning behaviors. We go on to show that MAG triggers a PTEN-dependent decrease in intrinsic growth cone PIP_3_ levels. Furthermore, the MAG-induced negative remodeling of growth cone β1-integrin adhesions is abolished by down-regulating PTEN expression. In contrast, MAG exposure in the presence of elevated cAMP signaling activity induced β1-integrin clustering in a manner similar to brain-derived neurotrophic factor (BDNF) and was PTEN-independent. These findings are, to our knowledge, the first demonstration that PTEN mediates chemorepulsion, acting mechanistically to suppress PIP_3_ signaling and negatively regulate integrins. Targeting these downstream actions may serve to promote regenerative nerve growth while permitting attractive guidance, leading to functional re-connectivity of neural circuits.

## EXPERIMENTAL PROCEDURES

### 

#### 

##### Down-regulating PTEN Expression

A morpholino designed to target the translational start site of *Xenopus* PTEN (5′-CGAACTCCTTGATGATGGCGGTCAT-3′, PTEN-MO; Gene Tools, LLC) was validated previously to down-regulate PTEN expression (25). The PTEN-MO (250 μm) was delivered by early embryo injection (2–4-cell stage; 10 ng/embryo) as described previously (13), which permits targeting CNS tissue. The dosage of PTEN-MO was titrated to minimize developmental defects. Only morphologically normal embryos were used for experiments. Specificity of the effects of PTEN-MO was validated by co-injecting a morpholino-resistant wild-type PTEN mRNA at the time of PTEN-MO injection. In growth cone turning assays, the PTEN-MO was conjugated to fluorescein and co-injected with Alexa Fluor 488-conjugated dextran for verification using fluorescence microscopy.

##### Primary Culture of Xenopus Spinal Neurons

We maintained wild-type *Xenopus laevis* (Nasco and Xenopus I) in approved animal facilities (Mayo Clinic) according to institutional and National Institutes of Health (Bethesda, MD) guidelines for animal care and safety. *In vitro* fertilization and dissociated primary neuron culture from stage 22 embryos of either sex were described previously (3, 13, 26). We plated spinal neurons onto laminin (25 μg/ml)-coated coverglass 14 h prior to experimentation, thus permitting robust functional testing by growth cone turning assays (3, 13, 26). Reagents were from Sigma unless indicated otherwise.

##### Quantitative Immunofluorescence Analysis of Akt Function

Cultured spinal neurons were fixed with 4% formaldehyde, permeabilized with 0.1% Triton X-100, and labeled for immunofluorescence microscopy with a primary antibody against phospho-Akt substrate (10 μg/ml, Cell Signaling Technology, 9611) and an appropriate Alexa Fluor 555-labeled secondary antibody (4 μg/ml; Invitrogen), as validated previously (13). Quantitative image analysis (ImageJ; National Institutes of Health) determined the mean thresholded fluorescence intensity within a region of interest containing the growth cone. All values were normalized to the control condition.

##### Quantitative Growth Cone Turning Assay

Growth cone turning assays were conducted as described previously (4). The micropipettes contained MAG (150 μg/ml; R&D Systems) or BDNF (50 μg/ml; PeproTech). Cultures were pretreated with cyclic nucleotide analogs ((*R*_p_)-cAMPS, 20 μm and (*S*_p_)-cAMPS, 20 μm; Merck) 30 min prior to the start of the turning assays. We monitored neurite growth for 15 min to determine the initial direction of extension, and the micropipette was positioned at a 45° angle relative to this axis. After 30 min, we measured the change in direction of extension relative to the initial trajectory.

##### Live Cell PIP_3_ Biosensor Imaging

We injected embryos at the 2–4-cell stage with DNA encoding the PIP_3_ biosensor PH_Akt_-GFP (27) (250 ng/ml; T. Balla, National Institutes of Health) and rhodamine dextran (250 mm; Invitrogen) as a volume reference as described previously (13). Some embryos were also injected with the PTEN-MO (250 μm; Gene Tools) as noted in the figure legends. We collected images at 15-s intervals throughout the experiment, starting 3 min prior to treatment with MAG (150 ng/ml, R&D Systems). Overexpression of PH_Akt_-GFP can act as a dominant negative inhibitor of Akt function, which precludes its utility for functional assays (13, 28). We chose growth cones with low PH_Akt_-GFP expression that had maintained filopodial activity for the imaging experiments. For image analysis (ImageJ, National Institutes of Health), we used a post-acquisition digital gain to adjust the rhodamine fluorescence intensity values to be 115% of the PH_Akt_-GFP intensity values in the growth cone central domain during the 3-min pretreatment period. We next subtracted the rhodamine intensity values from the PH_Akt_-GFP values on a pixel-by-pixel basis to eliminate the cytoplasmic signal that did not represent PIP_3_, which is a plasmalemmal lipid. We then thresholded the minimum gray level to background levels of the subtracted PH_Akt_-GFP images and measured the mean fluorescence intensity for pixels in a region of interest drawn around the growth cone (3, 13, 28). These values were then reported as the fluorescence intensity (*F*) normalized to the mean fluorescence intensity during the 3-min pretreatment period (*F*/*F*_0_) and may under-represent the magnitude of fluorescence change due to the presence of residual background signal. In the movie displays, post-acquisition images were set at a standard minimum threshold value to reduce more of the background signal, which may over-represent the magnitude of fluorescence change.

##### Integrin Immunofluorescence and Quantitative Analysis

Experiments on integrin surface levels and clustering were conducted as described previously (11, 29). Data were background-subtracted and normalized to appropriate control images. For quantification of integrin receptor clustering, we utilized a 3-fold fluorescence inclusion criterion of β1-integrin puncta intensity over the mean fluorescence of the growth cone central domain, as has been validated previously (29).

##### Statistical Analysis

Data were analyzed with GraphPad Prism software (version 5). The figure legends state the statistical tests used. Statistical comparisons of turning assay experiments utilized the Mann-Whitney *U* test due to the nonparametric distribution of the data. We used a repeated measures two-way ANOVA to compare the different imaging groups in Fig. 3. For all other data with a normal distribution, statistical comparisons utilized the two-tailed Student's *t* test. All integrin fluorescence data were normally distributed (D'Agostino and Pearson omnibus normality test) and were assessed using repeated measures one-way ANOVA with a Tukey's post hoc analysis.

## RESULTS AND DISCUSSION

**FIGURE 1.**
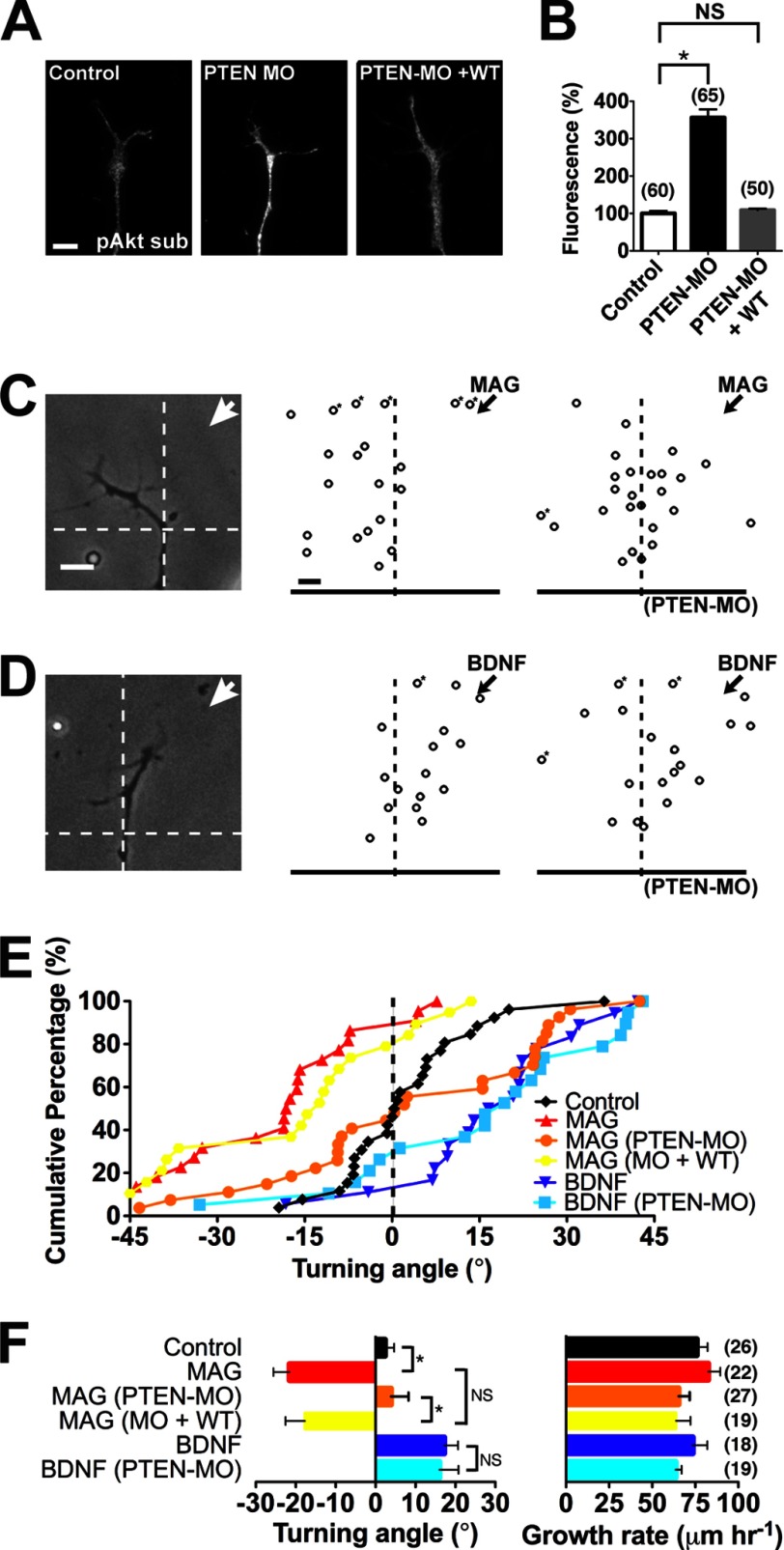
**Down-regulating PTEN selectively blocks MAG-induced repulsion.** *A*, *Xenopus* growth cones immunostained for Akt substrate phosphorylation (*pAkt sub*) under control conditions, with PTEN-MO alone and PTEN-MO plus wild-type rescue (*PTEN-MO* + *WT*). *Scale bar*, 5 μm. *B*, quantification of relative Akt substrate phosphorylation immunofluorescence normalized to the control condition. *C* and *D*, example images and summary plots depict the final position of wild-type and PTEN-MO growth cones relative to the starting position (origin) after 30 min of exposure to a gradient (*arrows*) of MAG (*C*) or BDNF (*D*). *Scale bar*, 10 μm. *Asterisks* denote growth outside of the plot. *E*, cumulative distribution of growth cone turning angles in response to a gradient of medium (*Control*), MAG, or BDNF either alone or with PTEN-MO and PTEN-MO plus wild-type PTEN (*MO* + *WT*). *F*, mean turning angles and growth rates from all experiments. All data are expressed as mean ± S.E. (*n* = number associated with each bar; *, *p* < 0.05; *NS*, no significant difference, *t* test (*B*) and Mann-Whitney *U* test (*F*)).

We down-regulated PTEN expression in the CNS by injecting a previously validated antisense morpholino oligonucleotide (PTEN-MO) into early *Xenopus* embryos (25). Immunostaining confirmed elevated Akt substrate phosphorylation in spinal neuron growth cones as compared with uninjected controls, indicating lower PTEN levels (Fig. 1, *A* and *B*). Injecting the PTEN-MO together with mRNA for a morpholino-resistant wild-type PTEN restored phospho-Akt substrate to control levels (Fig. 1, *A* and *B*). The spinal cord tissue, neuron number, and morphology of morphant and rescue neurons appeared similar to uninjected controls (data not shown but see Fig. 1*A*). To determine the function of PTEN in axonal growth cone chemotaxis, we utilized a quantitative bidirectional growth cone turning assay (Fig. 1, *C–F*). Applying a localized microscopic gradient of MAG (150 μg/ml) from a calibrated micropipette induced significant chemorepulsion of wild-type growth cones as compared with a control solution (Fig. 1, *C*, *E*, and *F*) as reported previously (3, 11, 30). When PTEN function was down-regulated (PTEN-MO), the MAG-induced chemorepulsion was abolished, and growth cones extended with no preferential turning (Fig. 1, *C*, *E*, and *F*). Expressing the morpholino-resistant wild-type PTEN during concomitant down-regulation of endogenous PTEN by the PTEN-MO completely rescued the MAG-induced chemorepulsion (Fig. 1, *E* and *F*). In contrast, applying a gradient of BDNF (50 μg/ml) induced chemoattraction of wild-type growth cones that persisted after down-regulating PTEN expression (Fig. 1, *D–F*). The rate of axon extension remained unchanged by any of the treatment conditions (Fig. 1*F*). The attractive growth cone turning independent of PTEN is surprising because chemoattraction requires phosphoinositide 3-kinase (PI3K) activity (5) and asymmetric PIP_3_ signaling (13). Thus, asymmetric PI3K/Akt activation may be sufficient for attraction, whereas repulsion may require PTEN-dependent depression of PIP_3_ signaling. Alternatively, a distinct phosphoinositide phosphatase may participate in growth cone chemoattraction.

**FIGURE 2.**
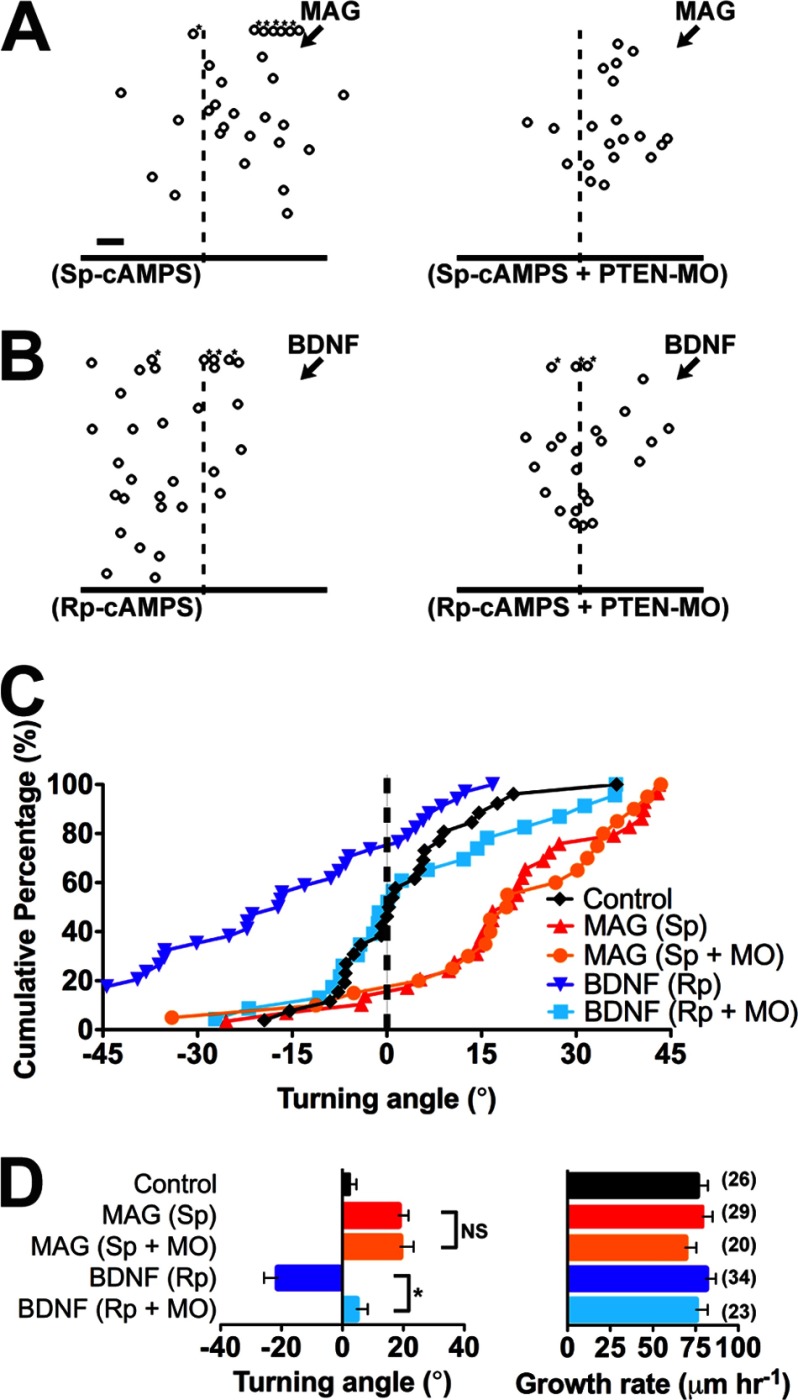
**Differential function of PTEN during cAMP-dependent conversion of turning responses.** *A* and *B*, summary plots depict the final position of wild-type and PTEN-MO growth cones relative to the starting position (origin) after 30 min of exposure to a gradient of MAG (*A*) and BDNF (*B*) in the presence of (*S*_p_)-cAMPS and (*R*_p_)-cAMPS, respectively. *Scale bar*, 10 μm. *Asterisks* denote growth outside of the plot. *C*, cumulative distribution of growth cone turning angles for all experiments as in *A* and *B. D*, mean turning angles and growth rates for all experiments. All data are mean ± S.E. (*n* = number associated with each bar; *, *p* < 0.05; *NS*, no significant difference, Mann-Whitney *U* test). The control group in *C* and *D* is the same as in Fig. 1, *E* and *F*.

Attractive and repulsive growth cone responses might be mechanistically related because a competitive cAMP analog converts chemoattraction induced by a BDNF gradient on *Xenopus* spinal neuron growth cones to chemorepulsion (31). Moreover, elevating cAMP signaling converts chemorepulsion induced by a MAG gradient to chemoattraction (30). We used this cyclic nucleotide-dependent modulation of growth cone chemotaxis to test the function of PTEN in MAG-induced chemoattraction (Fig. 2, *A–D*). Consistent with previous findings (30), when the membrane-permeable active cAMP analog (*S*_p_)-cAMPS was added to the bath saline (20 μm), the chemorepulsion normally induced by a MAG gradient converted to chemoattraction (Fig. 2, *A*, *C*, and *D*). The MAG-induced growth cone chemoattraction in the presence of (*S*_p_)-cAMPS persisted after down-regulating PTEN expression with the PTEN-MO (Fig. 2, *A*, *C*, and *D*). In contrast, growth cone chemorepulsion induced by a gradient of BDNF in the presence of the cAMP antagonist (*R*_p_)-cAMPS (20 μm) was abolished after down-regulating PTEN expression (Fig. 2, *B–D*). The rate of axon outgrowth was unaffected by any treatment condition (Fig. 2*D*). Uniquely, these findings demonstrate that PTEN function may be required for chemorepulsion in general rather than mediating actions of a particular chemotropic cue. Moreover, growth cone chemoattraction is PTEN-independent.

**FIGURE 3.**
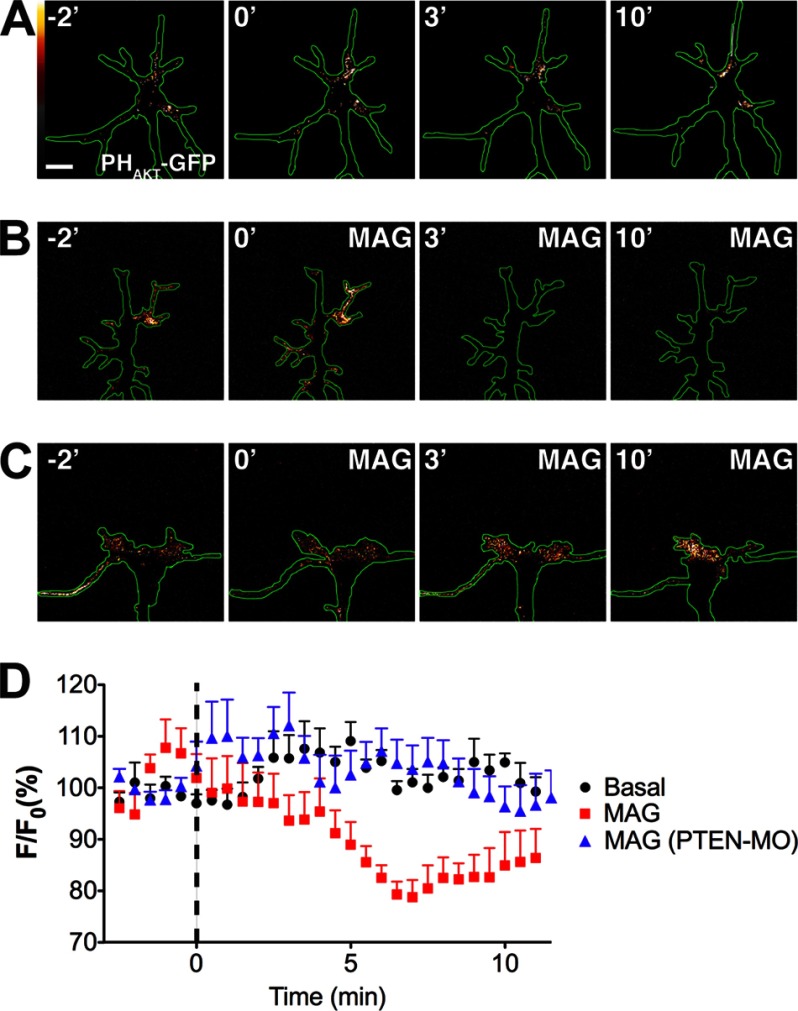
**MAG induces PTEN-dependent depression of PIP_3_ levels.** *A–C*, pseudocolor time-lapse confocal images of PH_Akt_-GFP standard-corrected for growth cone thickness during basal conditions (*A*, *n* = 6; see also supplemental Movie S1) and with MAG treatment (150 ng/ml; starting at time = 0) either alone (*B*, *n* = 6; see also supplemental Movies S2–S4) or after down-regulating PTEN expression (*C*, PTEN-MO, *n* = 7; see also supplemental Movie S5). Time is in minutes. *White* is highest fluorescence intensity. *Scale bar*, 5 μm. *D*, quantification of change in fluorescence intensity as compared with the mean intensity prior to treatment (*F*/*F*_0_). Treatment groups are significantly different (*p* = 0.0029, repeated measures two-way ANOVA).

The PTEN-dependent axon regrowth after injury requires translational reprogramming (22), but the function of PTEN in actively extending growth cones is unknown. We hypothesized that PTEN also mediates the potent inhibitory outgrowth effects of MAG by locally decreasing PIP_3_ levels required for growth cone extension. To determine whether intrinsic PIP_3_ levels are depressed by exposure to MAG, we utilized live cell imaging of a PIP_3_ biosensor comprising the PIP_3_-binding pleckstrin homology domain of Akt fused to GFP (PH_Akt_-GFP) (13, 27). Expressing PH_Akt_-GFP together with an injected fluorescent volume standard (rhodamine-dextran) permitted correction for differences in growth cone thickness (PH_Akt_-GFP-rhodamine) and revealed the basal PIP_3_ levels in growth cones extending on a laminin substrate (Fig. 3*A*). The basal PIP_3_ levels generally were concentrated within the peripheral domain and fluctuated stochastically, but persisted throughout the imaging period (Fig. 3*A* and supplemental Movie S1). In contrast, growth cone PIP_3_ levels decreased globally within 3–5 min following bath application of MAG (150 ng/ml) relative to the pretreatment period (Fig. 3*B* and supplemental Movies S2–S4). Quantification of the standard-corrected mean fluorescence intensity for all growth cones revealed significant depression of PIP_3_ levels in the presence of MAG as compared with untreated growth cones, consistent with increased PTEN phosphoinositide phosphatase activity (Fig. 3*D*). Down-regulating PTEN expression abolished the MAG-induced depression of growth cone PIP_3_ levels, which remained unchanged during MAG treatment relative to the pretreatment period (Fig. 3, *C* and *D*, and supplemental Movie S5). Taken together, these findings support the notion that basal PIP_3_ levels correlate positively with conditions permissive for axon outgrowth and that the inhibitory action of MAG induces PTEN-dependent depression of PIP_3_ levels locally in the growth cone.

**FIGURE 4.**
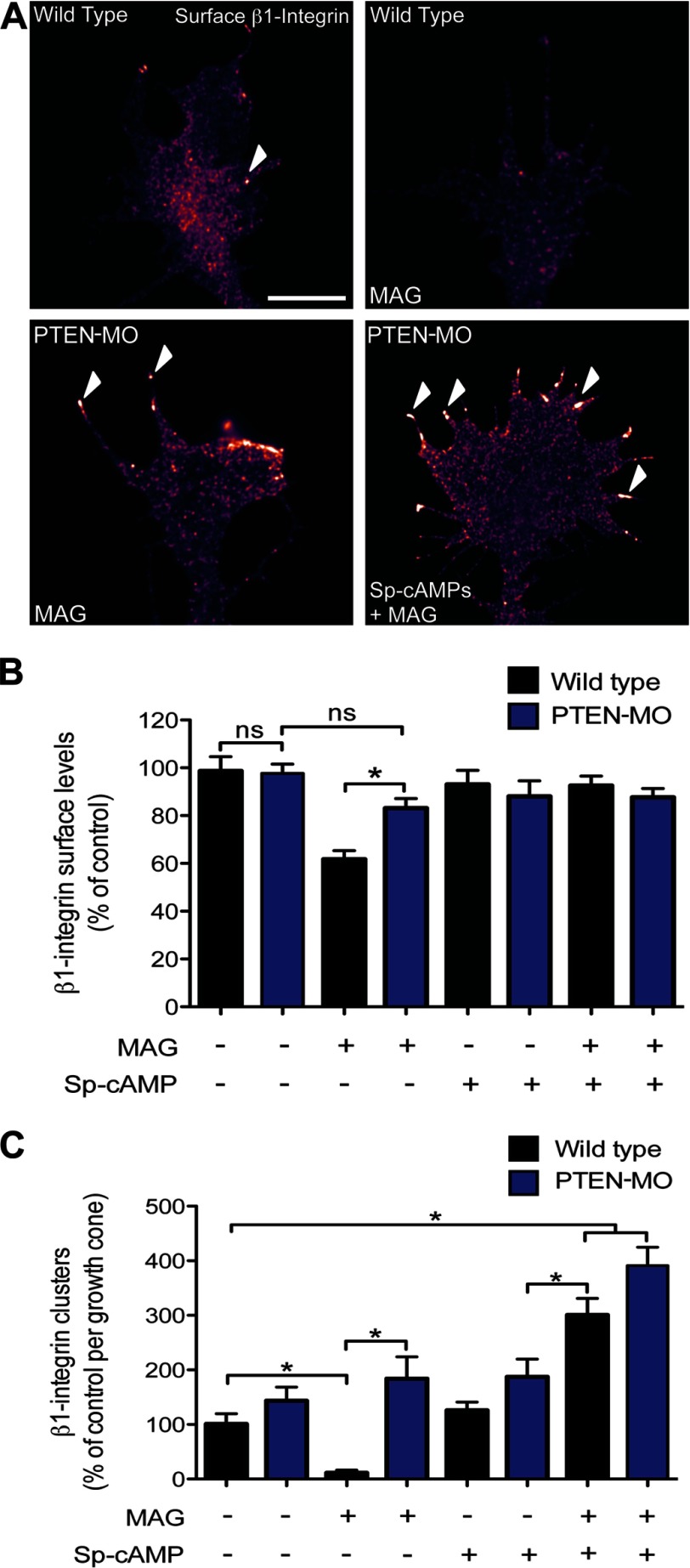
**MAG-induced β1-integrin internalization and inhibition of clustering is PTEN-dependent.** *A*, representative immunofluorescence images show the distribution of β1-integrin in the growth cone of wild-type and PTEN-MO neurons after experimental treatments: control (BSA), MAG (1 μg/ml; 5 min), or (*S*_p_)-cAMPS (20 μm) + MAG. *Arrowheads* designate clustered β1-integrin. *Scale bar*, 5 μm. *B* and *C*, summary graphs show the quantification of β1-integrin surface levels (*B*) and β1-integrin clustering (*C*) for all experimental conditions. Data are the mean ± S.E. (*n* > 150, *, *p* < 0.05, one-way ANOVA with a Tukey's post hoc analysis).

Chemorepulsion by MAG requires endocytic removal of β1-integrin receptors from the growth cone plasmalemma and negative remodeling of integrin adhesions (11). Interestingly, PTEN activity has been shown to inhibit cell migration by negatively regulating integrin-based focal adhesions (32, 33). We therefore investigated the potential role of PTEN in regulating integrin function in the growth cone. Consistent with previous findings (11, 29), surface immunostaining and quantitative analysis demonstrated removal of global β1-integrin and loss of clustered β1-integrin from the plasmalemma of wild-type growth cones after MAG application (Fig. 4, *A–C*). In growth cones with down-regulated PTEN expression (PTEN-MO), the MAG-induced removal of both global and clustered β1-integrin was blocked, and surface levels and spatial distribution were similar to wild-type untreated controls (Fig. 4, *A–C*). Preincubation with (*S*_p_)-cAMPS also abolished the MAG-induced loss of global surface β1-integrin (Fig. 4, *A* and *B*). Intriguingly, neurons that received the (*S*_p_)-cAMPS pretreatment followed by MAG exposure demonstrated increased β1-integrin clustering in the growth cone periphery (Fig. 4, *A* and 4*C*). These observations are consistent with previous findings reporting positive remodeling of β1-integrin receptors in the growth cone induced by an attractive cue (29). The MAG-induced increase in β1-integrin clustering in the presence of (*S*_p_)-cAMPS occurred independently of PTEN (PTEN-MO) (Fig. 4*C*). Thus, the differential function of PTEN in negatively regulating β1-integrin levels and clustering correlates with repulsive *versus* attractive growth conditions.

In this study, we examined the function of PTEN in chemotactic growth cone guidance. Our results indicate that PTEN activity is a selective mediator of repulsive growth cone guidance, but is not required for growth cone chemoattraction. The repulsive action of PTEN correlates with local phosphoinositide phosphatase activity because the basal PIP_3_ levels in growth cones extending on a permissive laminin substrate were depressed significantly by MAG treatment. Down-regulating PTEN expression also revealed a distinct role for PTEN in the negative remodeling of integrin adhesions at the growth cone surface membrane. Taken together, these findings indicate that chemorepellents potentiate PTEN activity, which decreases PIP_3_ levels and negatively regulates integrins. Importantly, down-regulating PTEN has shown great promise in promoting regenerative axon outgrowth after injury. The present results complement these prior studies and, collectively, suggest that modulating PTEN expression would permit attractive axon guidance while repressing chemorepulsion by local inhibitory cues.

## Footnotes

3 The abbreviations used are:

PIP_3_: phosphatidylinositol 3,4,5-triphosphate
MAG: myelin-associated glycoprotein
MO: morpholino oligonucleotide
PH_Akt_-GFP: Pleckstrin homology domain of Akt fused to GFP
PTEN: phosphatase and tensin homologue
ANOVA: analysis of variance
(*R*_p_)-cAMP: adenosine-3′,5′-cyclic monophosphorothioate, *R*_p_ isomer
(*S*_p_)-cAMP: adenosine-3′,5′-cyclic monophosphorothioate, *S*_p_ isomer.
